# Genetic Liabilities Differentiating Bipolar Disorder, Schizophrenia, and Major Depressive Disorder, and Phenotypic Heterogeneity in Bipolar Disorder

**DOI:** 10.1001/jamapsychiatry.2022.2594

**Published:** 2022-08-31

**Authors:** Alexander L. Richards, Alastair Cardno, Gordon Harold, Nicholas J. Craddock, Arianna Di Florio, Lisa Jones, Katherine Gordon-Smith, Ian Jones, Ruth Sellers, James T. R. Walters, Peter A. Holmans, Michael J. Owen, Michael C. O’Donovan

**Affiliations:** 1 MRC Centre for Neuropsychiatric Genetics and Genomics, Division of Psychological Medicine and Clinical Neurosciences, School of Medicine, Cardiff University, Cardiff, United Kingdom; 2 Leeds Institute of Health Sciences, Division of Psychological and Social Medicine, Faculty of Medicine and Health, University of Leeds, Leeds, United Kingdom; 3 Faculty of Education, University of Cambridge, Cambridge, United Kingdom; 4 School of Medicine, Child and Adolescent Psychiatry Unit, University College Dublin, Ireland; 5 Department of Primary Care & Public Health, Brighton & Sussex Medical School, University of Sussex, Brighton, United Kingdom; 6 Psychological Medicine, University of Worcester, Worcester, United Kingdom

## Abstract

**Importance:**

Understanding the origins of clinical heterogeneity in bipolar disorder (BD) will inform new approaches to stratification and studies of underlying mechanisms.

**Objective:**

To identify components of genetic liability that are shared between BD, schizophrenia, and major depressive disorder (MDD) and those that differentiate each disorder from the others and to examine associations between heterogeneity for key BD symptoms and each component.

**Design, Setting, and Participants:**

Using data from the Bipolar Disorder Research Network in the United Kingdom, components of liability were identified by applying genomic structural equation modeling to genome-wide association studies of schizophrenia, BD, and MDD. Polygenic risk scores (PRS) representing each component were tested for association with symptoms in an independent BD data set. Adults with *DSM*-*IV* BD or schizoaffective disorder, bipolar type, were included. Data were collected from January 2000 to December 2013, and data were analyzed from June 2020 to February 2022.

**Main Outcomes and Measures:**

PRS representing the components of liability were tested for association with mania and depression, psychosis, and mood incongruence of psychosis in participants with BD, measured using the Bipolar Affective Disorder Dimensional Scale.

**Results:**

Of 4429 included participants, 3012 (68.0%) were female, and the mean (SD) age was 46.2 (12.3) years. Mania and psychosis were associated with the shared liability component (mania β = 0.29; 95% CI, 0.23-0.34; *P* = 3.04 × 10^−25^; psychosis β = 0.05; 95% CI, 0.04-0.07; *P* = 2.33 × 10^−13^) and the components that differentiate each of schizophrenia (mania β = 0.08; 95% CI, 0.03-0.14; *P* = .002; psychosis β = 0.03; 95% CI, 0.01-0.04; *P* = 1.0 × 10^−4^) and BD (mania β = 0.14; 95% CI, 0.09-0.20; *P* = 1.99 × 10^−7^; psychosis β = 0.02; 95% CI, 0.01-0.03; *P* = .006) from the other disorders. The BD differentiating component was associated with mania independently of effects on psychosis (β = 0.14; 95% CI, 0.08-0.20; *P* = 4.32 × 10^−6^) but not with psychosis independently of mania. Conversely, the schizophrenia differentiating component was associated with psychosis independently of effects on mania (β = 0.01; 95% CI, 0.003-0.03; *P* = .02), but not with mania independently of psychosis. Mood incongruence of psychosis was associated only with the schizophrenia differentiating component (β = 0.03; 95% CI, 0.01-0.05; *P* = .005). Depression was associated with higher MDD differentiating component (β = 0.07; 95% CI, 0.01-0.12; *P* = .01) but lower BD differentiating component (β = −0.11; 95% CI, −0.17 to −0.06; *P* = 7.06 × 10^−5^).

**Conclusions and Relevance:**

In this study of BD, clinical heterogeneity reflected the burden of liability to BD and the contribution of alleles that have differentiating effects on risk for other disorders; mania, psychosis, and depression were associated with the components of genetic liability differentiating BD, MDD, and schizophrenia, respectively. Understanding the basis of this etiological heterogeneity will be critical for identifying the different pathophysiological processes underlying BD, stratifying patients, and developing precision therapeutics.

## Key Points

**Question:**

Are there etiological associations between clinical heterogeneity in participants with bipolar disorder and components of genetic liability that are shared between schizophrenia, bipolar disorder, and major depressive disorder and components that differentiate between these disorders?

**Findings:**

In this genetic association study including 4429 participants, mania, psychosis, and depression were associated with the components of genetic liability differentiating bipolar disorder, major depressive disorder, and schizophrenia, respectively. The shared liability component was associated with mania.

**Meaning:**

This study advances understanding of etiological heterogeneity in individuals with bipolar disorder by showing clinical heterogeneity of bipolar disorder is underpinned by etiological heterogeneity linked to components of differentiating genetic liability that reflects the symptomatology of the cognate disorders.

## Teaser

This genetic association study identifies components of genetic liability that are shared between bipolar disorder (BD), schizophrenia, and major depressive disorder (MDD) and those that that differentiate each disorder from the others.

## Introduction

Bipolar disorder (BD) is highly heritable (70% to 90%).^1,2^ Genome-wide association studies (GWASs) suggest that thousands of common risk alleles are involved and that such variants account for around 20% of variance in liability to the disorder,^3,4^ although the incomplete coverage of GWAS arrays means this is likely to be underestimated. Other contributions to genetic liability to BD come from rare copy number variants and deleterious coding variants; how much these contribute to liability is unclear.^5,6^

BD is heterogeneous and its symptoms overlap with other psychiatric disorders, particularly major depressive disorder (MDD) and schizophrenia. While a history of 1 or more periods of mania or hypomania is always present, other features vary, including the presence and severity of depressive and psychotic symptoms. Psychotic symptoms are further classified as mood-incongruent psychotic symptoms (MIPS) when they appear inconsistent with mood state. Liability to BD overlaps with liability to MDD and schizophrenia—that is, risk alleles are pleiotropic. Common variant liability to BD has a genetic correlation with that for schizophrenia (*rg* of approximately 0.7) and MDD (*rg* of approximately 0.45).^4^ Liability to MDD and schizophrenia also overlap (*rg* of approximately 0.4).^7^ These genetic correlations may partly explain the increased risk of BD in the families of probands with schizophrenia and MDD.^8^

The substantial but incomplete pleiotropy implies that individuals with BD differ not only by their total burden of risk alleles for BD but also by the extent to which they carry risk alleles that are pleiotropic for schizophrenia and depression and for those that do not confer risk to either of those disorders. It has been proposed that clinical overlaps between disorders as well as phenotypic heterogeneity within BD might reflect these genetic differences.^9^ Consistent with this hypothesis, among individuals with BD, schizophrenia liability as indexed by polygenic risk score (PRS) is higher in people with mania^10^ and also psychosis,^11^ particularly MIPS.^12^ However, interpreting these findings is complicated by genetic correlations between traits, because among individuals with BD, people with higher liability to schizophrenia also tend to have higher liability to BD.

Here, we sought to further examine the hypothesis that phenotypic heterogeneity within BD might be correlated with genetic heterogeneity. Using data from GWAS data sets of BD, schizophrenia, and MDD, we used genomic structural equation modeling (gSEM)^13^ to isolate those components of liability that distinguish disorders from one another as well as the pleiotropic component that is shared between the disorders. gSEM has previously been used to identify shared and nonshared genetic liability between autism and attention-deficit/hyperactivity disorder,^14^ depression and anxiety,^15^ and educational attainment and IQ.^16^ We then tested the various components of liability for their associations with some of the key symptoms of BD, specifically, psychosis, MIPS, mania, and depression. Overall, our findings support the hypothesis that within BD, the clinical picture presented by each individual is influenced by not only their genetic liability to BD but also by alleles with shared effects on and relatively specific effects for other disorders.

## Methods

### Source GWAS

The 3 source GWAS for gSEM were of schizophrenia, BD, and MDD^4,17,18^ conducted by the Psychiatric Genomics Consortium (PGC) (eTable 1 in the Supplement). The BD GWAS included our target BD data set; to ensure sample independence for PRS analysis, we obtained from the PGC a custom GWAS that excluded our sample.^4^ Source GWASs were restricted to European individuals. We did not include the 23andMe subset of the MDD source GWAS, as this used a broad definition of affected status, which could affect genetic associations between the disorders.^17^ The Bipolar Disorder Research Network study was given a favorable ethical opinion by the West Midlands Multi-Centre Research Ethics Committee. Local research and development approval was obtained in all participating National Health Service Trusts and Health Boards. All participants gave written informed consent.

We limited source GWASs to heritable single-nucleotide variants (SNVs) with a minor allele frequency greater than 1% in HapMap 3 (which gSEM uses as a reference set)^19^ and which were present in all source GWASs, with an imputation info score of 0.7 or greater. Variants within the extended MHC were excluded (chromosome 6 from 25 megabase to 35 megabase), leaving 6 929 980 SNVs.

### Genomic SEM

We used gSEM to apply a common factor model to the summary statistics from the source GWAS (eFigure 1A and eMethods in the Supplement). gSEM estimates and corrects for sample overlap among the source GWAS. For each SNV, the loading on the common factor was extracted to produce a statistic corresponding to what we term the *shared effect*, the effect size of that variant that is shared across the 3 source GWASs. We then applied 3 models where we extracted the loading of each SNV on the residual variance from each source GWAS that was not explained by the common factor (eFigure 1B-D in the Supplement), so that the residual effect sizes for each SNV index how much it influences the probability of having a particular phenotype (relative to the 2 other source phenotypes). We refer to these as schizophrenia differentiating, BD differentiating, and MDD differentiating components. We then applied genome-wide measures of shared and differentiating effects to test for associations between the components of liability and various clinical features of BD, using a PRS approach.^20^ SNV-based heritabilities were calculated using linkage disequilibrium score regression.^21,22^

See eMethods in the Supplement for details, including heritability calculations and a comment on power. gSEM was run in R version 4.0.3 (The R Foundation) using the GenomicSEM package.^13^

### Target Data Set and Phenotypes

The BD target data set for PRS analysis contained individuals with *DSM*-*IV* BD and schizoaffective disorder, bipolar type, recruited in the United Kingdom by the Bipolar Disorder Research Network (see eTables 2 to 4 in the Supplement for sample sizes, demographic characteristics, bipolar subtypes, and details of samples with psychosis and MIPS data).^23^ Lifetime psychotic symptomatology and MIPS were rated using the Bipolar Affective Disorder Dimensional Scale (BADDS) using the BADDS-P and BADDS-I subscales^24^ (eFigures 2 and 3 in the Supplement), representing ordinal measures of lifetime symptom domain severity with high interrater reliability.^24^ Psychosis and MIPS were analyzed as categorical variables. Psychosis was defined as the lifetime presence of clear-cut psychotic symptoms, corresponding to a BADDS-P score of 10 or more.^24^ Psychotic symptoms were considered mood incongruent when they occurred outside an affective episode or if they included thought echo, insertion, withdrawal, or broadcasting; passivity experiences; hallucinatory voices giving running commentary, discussing subject in third person, or originating in some part of the body; bizarre delusions; or catatonia. MIPS was considered present if at least as many mood-incongruent as mood-congruent psychotic symptoms were reported, corresponding to a BADDS-I score of 20 or more.^12,24^ BADDS-I was only rated in individuals who met the psychosis presence criterion.

Lifetime manic and depressive symptoms were rated using the BADDS-M and BADDS-D subscales, respectively (eFigures 4 and 5 in the Supplement), and analyzed as ordinal variables.^24^ Associations between the 4 ordinal BADDS scales were examined using polychoric correlation, and *P* values estimated by bootstrapping (100 000 iterations) using R packages polychor and cor.ci.

### Statistical Analysis

#### PRS Analyses

PRS were calculated for 4 sets of gSEM-derived summary statistics; shared liability, schizophrenia differentiating, BD differentiating, and MDD differentiating components, as described.^20^ To be conservative, we applied the original PRS methodology, as newer methods have not been validated for use in gSEM-derived components. Clumping was performed on imputed best-estimate genotypes for each GWAS using PLINK (maximum *r*^2^ = 0.2; window = 500 kb; minimum minor allele frequency = 0.1; minimum info score = 0.7). Optimal *P* value thresholds for including alleles in gSEM-derived PRS are unknown, and since we cannot derive these in independent samples, we performed PRS analysis without *P* value thresholding.

We tested PRS for association using logistic regression for dichotomous variables (psychosis and MIPS) and ordinal logistic regression for ordinal variables (BADDS-M and BADDS-D scores), reporting β and *P* values for the PRS term in the regression model. Statistical significance was set at *P* < .05, and all *P* values were 2-tailed. Association analyses were adjusted for the first 10 population principal components, age at interview, and genotyping platform.^23^ Statistical analyses were conducted in R. All PRS variables were standardized before analysis using the scale() function in R.

#### Sensitivity Analyses 

We used ordinal logistic regression to test the ordinal BADDS-P and BADDS-I scores for associations to ensure that thresholding to produce dichotomous variables did not affect our results. To examine the effects of including participants with schizophrenia, bipolar type, we repeated the association analyses with schizophrenia, bipolar type, samples excluded. Correlations between gSEM PRS were examined using Pearson correlation.

## Results

### Heritability and Correlations

Of 4429 included participants, 3012 (68.0%) were female, and the mean (SD) age was 46.2 (12.3) years. SNV heritability values for the gSEM components are given in Table 1, and Pearson correlations for the PRS derived from each source GWAS and gSEM component are given in eTable 5 in the Supplement. Source GWAS PRS were positively correlated with the shared liability fraction PRS and with their corresponding differentiating fraction PRS. Correlations between differentiating component PRS and shared liability PRS were small (*r* range, −0.1 to 0.1).

**Table 1.** Heritability of Genomic Structural Equation Modeling–Produced Summary Statistics

PRS	SNV Heritability (SE)
Shared	0.300 (0.009)
Schizophrenia differentiating	0.155 (0.008)
BD differentiating	0.099 (0.007)
MDD differentiating	0.092 (0.006)

Abbreviations: BD, bipolar disorder; MDD, major depressive disorder; PRS, polygenic risk score; SNV, single-nucleotide variability (observed scale).

BADDS-P psychosis scores were moderately positively correlated with BADDS scores for mania (*r* = 0.61) and MIPS (*r* = 0.38) but weakly correlated with depression scores (*r* = 0.07). Other phenotype pairs were weakly correlated (*r* range, −0.06 to 0.14; eTable 6 in the Supplement).

### Psychosis and MIPS

**Figure 1.**
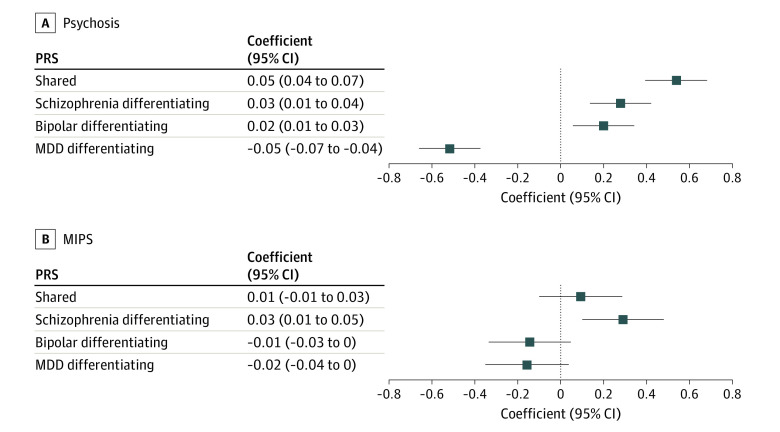
Association Between Psychosis and Mood-Incongruent Psychotic Symptoms (MIPS) and Polygenic Risk Scores Representing Genomic Structural Equation Modeling Fractions of Liability MDD indicates major depressive disorder.

Psychosis in BD was associated with higher shared liability (β = 0.05; 95% CI, 0.04-0.07; *P* = 2.33 × 10^−13^), schizophrenia differentiating liability (β = 0.03; 95% CI, 0.01-0.04; *P* = 1.0 × 10^−4^), and BD differentiating liability (β = 0.02; 95% CI, 0.01-0.03; *P* = .006) but with lower MDD differentiating liability (β = −0.05; 95% CI, −0.007 to −0.04; *P* = 1.26 × 10^−12^) (Figure 1; eTable 7 in the Supplement). However, associations with MIPS showed a different picture; we found significant evidence for association only with the schizophrenia differentiating component (β = 0.03; 95% CI, 0.01-0.05; *P* = .005), with a similar effect size to that between the schizophrenia differentiating component and psychosis as a whole. The findings for the shared fraction of liability are particularly notable. Despite this fraction being the strongest and by far the most highly significant predictor of unstratified psychosis, it was not significantly associated with MIPS. We found no evidence that higher BD differentiating liability was associated with MIPS, although the confidence interval means we cannot exclude weak effects on increasing risk.

Similar patterns were obtained when psychosis and MIPS were treated as ordinal variables (eTable 7 in the Supplement; note that the βs reported for ordinal analyses are not comparable with those from the dichotomous psychosis and MIPS analyses). Excluding participants with schizophrenia, bipolar type, had minimal effect (eTable 8 in the Supplement).

### Mania and Depression

**Figure 2.**
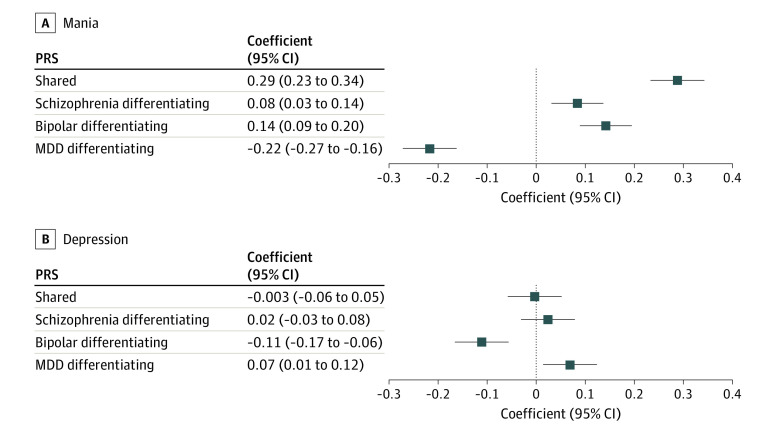
Association Between Bipolar Affective Disorder Dimensional Scale Mania and Depression Scores and Polygenic Risk Scores Representing Genomic Structural Equation Modeling Fractions of Liability MDD indicates major depressive disorder.

Increased mania scores in BD were associated with higher shared PRS (β = 0.29; 95% CI, 0.23-0.34; *P* = 3.04 × 10^−25^), schizophrenia differentiating PRS (β = 0.08; 95% CI, 0.03-0.14; *P* = .002), and BD differentiating PRS (β = 0.14; 95% CI, 0.09-0.20; *P* = 1.99 × 10^−7^) and lower MDD differentiating PRS (β = −0.22; 95% CI, −0.27 to −0.16; *P* = 2.84 × 10^−15^) (Figure 2; eTable 9 in the Supplement), a picture similar to that for associations between the gSEM PRS and psychosis. Increased depression scores in BD were associated with higher MDD differentiating PRS (β = 0.07; 95% CI, 0.01-0.12; *P* = .01) and lower BD differentiating PRS (β = −0.11; 95% CI, −0.17 to −0.06; *P* = 7.06 × 10^−5^) (Figure 2; eTable 9 in the Supplement). We found no evidence of an association with shared liability or schizophrenia differentiating PRS. Excluding participants with schizophrenia, bipolar type, had little effect (eTable 10 in the Supplement).

### Disentangling Effects on Mania and Psychosis

Mania and psychosis were correlated in our sample and showed similar patterns of PRS associations. To identify independent effects of genetic liability on these phenotypes, we repeated the analyses of psychosis using mania as a covariate (Table 2) and mania using psychosis as a covariate (Table 3). The effects of the shared and BD differentiating components on psychosis were not significant after conditioning on mania, while that of the schizophrenia differentiating fraction remained significant (β = 0.01; 95% CI, 0.003-0.03; *P* = .02). In contrast, after conditioning on psychosis, schizophrenia differentiating component was not significantly associated with mania, while the associations of mania with the shared component (β = 0.23; 95% CI, 0.17-0.29; *P* = 6.26 × 10^−4^), BD differentiating component (β = 0.14; 95% CI, 0.08-0.20; *P* = 4.32 × 10^−6^), and the MDD differentiating component (β = −0.13; 95% CI, −0.19 to −0.07; *P* = 1.21 × 10^−5^) remained highly significant.

**Table 2.** Logistic Regression of Genomic Structural Equation Modeling Polygenic Risk Score (PRS) on Psychosis (Unconditioned and Conditioned on Mania)^a^

PRS	Psychosis		Psychosis (conditioned on mania)	
	β (95% CI)	*P* value	β (95% CI)	*P* value
Shared	0.054 (0.040 to 0.069)	2.33 × 10^−13^	0.009 (−0.003 to 0.02)	1.33 × 10^−1^
Schizophrenia differentiating	0.028 (0.014 to 0.042)	1.00 × 10^−4^	0.014 (0.003 to 0.030)	1.65 × 10^−2^
BD differentiating	0.020 (0.006 to 0.034)	6.00 × 10^−3^	−0.004 (−0.015 to 0.007)	4.79 × 10^−1^
MDD differentiating	−0.052 (−0.066 to −0.038)	1.26 × 10^−12^	−0.013 (−0.025 to −0.002)	2.41 × 10^−2^

Abbreviations: BD, bipolar disorder; MDD, major depressive disorder.

^a^ Analyses include age at interview, genotyping platform, and 10 population principal components as covariates. Note that βs are on different scales in Tables 2 and 3 and are not comparable.

**Table 3.** Ordinal Logistic Regression of Genomic Structural Equation Modeling Polygenic Risk Score (PRS) With Mania (Unconditioned and Conditioned on Psychosis)^a^

PRS	Mania		Mania (conditioned on psychosis)	
	β (95% CI)	*P* value	β (95% CI)	*P* value
Shared	0.288 (0.233 to 0.342)	3.04 × 10^−25^	0.232 (0.172 to 0.293)	6.26 × 10^−14^
Schizophrenia differentiating	0.084 (0.031 to 0.138)	2.00 × 10^−3^	−0.004 (−0.063 to 0.055)	8.94 × 10^−1^
BD differentiating	0.142 (0.089 to 0.196)	1.99 × 10^−7^	0.139 (0.080 to 0.198)	4.32 × 10^−6^
MDD differentiating	−0.217 (−0.271 to −0.163)	2.84 × 10^−15^	−0.134 (−0.194 to −0.074)	1.21 × 10^−5^

Abbreviations: BD, bipolar disorder; MDD, major depressive disorder.

^a^ Analyses include age at interview, genotyping platform, and 10 population principal components as covariates. Note that βs are on different scales in Tables 2 and 3 and are not comparable.

## Discussion

Our study was motivated by clinical heterogeneity within psychiatric diagnoses, cross-disorder overlaps in their clinical features, and genetic findings consistent with widespread pleiotropic effects of risk alleles. Our hypothesis was that these observations are related, and specifically that clinical heterogeneity in BD reflects not only the total liability for BD carried by an individual but also the composition of that liability in terms of alleles that are specific to BD, those that have pleiotropic effects on other disorders, and alleles that have relatively specific effects on risk for other disorders. To examine this, we used gSEM to derive from GWAS of schizophrenia, BD, and MDD a component of liability that is shared across the disorders as well as components that differentiate each disorder from the others. We then used PRS based on these components to examine their associations with psychosis, MIPS, and severity of manic and depressive symptoms in individuals with BD.

The patterns of association between symptoms and components of genetic liability that differentiate between disorders largely reflect the characteristic symptomatology of the cognate disorder. MIPS (a characteristic of schizophrenia) was associated only with higher schizophrenia differentiating liability while the higher severity of depression was associated with higher MDD differentiating PRS. The patterns of association for psychosis and mania were more complex, as both were associated with increased liability that was specific to each of schizophrenia and BD. However, severity of mania and psychosis were moderately correlated in our sample, likely due at least in part to psychosis being one of the impairment criteria that distinguishes hypomania from mania.^24^ We therefore sought to tease apart effects on psychosis and mania using conditional analyses and found that higher BD differentiating liability was associated with severity of mania (the characteristic feature of BD) independently of the presence of psychosis but not with psychosis independent of mania severity. In contrast, the schizophrenia differentiating component was associated with psychosis (the most characteristic feature of schizophrenia) independent of mania severity but not mania severity independent of psychosis. These findings are again consistent with the idea that the patterns of association largely reflected the characteristic symptomatology of the cognate disorder. They also suggest that there are (at least) 2 partly distinct mechanisms underpinning manic symptoms: one related to the presence of psychosis and linked to alleles that are relatively specific for schizophrenia and one driven by severity of mania linked to alleles that are relatively specific for BD. Our finding is partly consistent with a 2011 systematic review of factor analysis approaches to bipolar symptoms,^11^ in which psychotic symptoms were present in 2 factors, one which included elevated mood and the other which did not. It is also consistent with evidence that, compared with individuals with nonpsychotic BD, those with psychotic BD have higher familial genetic liability to both BD and schizophrenia^25^ and that, in monozygotic twins, when probands have cooccurring mania and MIPS, their twins have elevated risk of both BD and schizophrenia.^26,27^

Higher shared liability was associated with psychosis and mania but not with MIPS or depression. The conditional analysis suggested a pattern similar to that observed for the BD differentiating fraction; higher shared liability was associated with mania independently of psychosis but was not associated with psychosis independently of mania. The lack of evidence for association between the shared liability component and both depression and MIPS is consistent with the relatively selective relationships between those 2 phenotypes and MDD differentiating and schizophrenia differentiating components, respectively.

We found instances where higher liability to a differentiating component was associated with reduced symptomatology, for example, higher liability to the MDD differentiating component was associated with absence of psychosis and lower severity of mania. It seems unlikely that, in general, alleles that increase liability to a major psychiatric disorder are, per se, protective against symptoms for another, although this may be true for specific alleles, as for instance has been seen in ulcerative colitis and Crohn disease.^28^ In the present study, such associations must be interpreted in the context of negative correlations between the differentiating factors. These imply that people with a higher MDD differentiating score will tend to have lower schizophrenia differentiating and BD differentiating scores and therefore lower liability to phenotypes that schizophrenia differentiating and BD differentiating components are associated with (in this example, psychosis and mania). However, we acknowledge our study does not formally exclude the possibility of true protective effects.

BD symptom severity, particularly of mania and presence of psychosis, has usually been considered to indicate more severe disorder. It has also been widely assumed that phenotypic severity is related to a higher burden of BD risk alleles.^9^ Our findings that gSEM components that index liability to BD (the shared and BD differentiating components) are associated with the severity of these symptoms are consistent with this view. However, the observations that MIPS and depressive symptoms were not associated with higher shared or BD differentiating liabilities components but were influenced by higher schizophrenia differentiating and MDD differentiating liabilities, respectively, point to a model where some symptoms within BD are influenced by genetic variation that is partially or wholly independent of BD liability.^9^

### Limitations

Our study has limitations. First, we were unable to access a replication data set, and therefore, confirmation of our findings is required. Second, while our finding that the differentiating liability components within BD were associated with symptoms that are characteristic of their respective source disorders suggests that those components might similarly influence symptomatology in other disorders,^11,12,29^ this hypothesis needs to be empirically tested in large, well-phenotyped samples of other disorders, particularly schizophrenia and MDD. Third, the relative lack of relevant data from people of non-European ancestries means that our results may not generalize beyond that population, underscoring the need to increase the population diversity in genomic studies.^18^ Fourth, the source GWAS and the target BD samples were not completely representative of the disorders at the population level, and this is likely to influence to some extent how liability is apportioned into the various shared and differentiating fractions. Moreover, given the high prevalence of MDD in the population and the widespread use of unscreened controls in genomic studies, it is likely that schizophrenia and BD source GWASs include controls who have MDD. In principle, the presence of individuals with MDD in the schizophrenia and BD GWAS study controls can be expected to reduce the effect size estimates of alleles shared with MDD, thereby reducing the influence of MDD on the shared gSEM-derived component, and somewhat inflating the MDD differentiating component. However, the net effects of this effect in the context of other ascertainment biases in the source GWAS are difficult to predict. Additionally, we note the GWAS arrays used to study common variation capture only a modest amount of the total heritability of these disorders (SNV heritability of 7% to 35%, observed scale). The genetic architecture underlying the unattributed heritability is not yet clear, but certainly includes uncommon and even very rare variants, some of fairly high penetrance. When sufficient data are available, it will be important to study how these additional sources of variation, together with relevant environmental exposures, influence phenotypic expression.

## Conclusions

In summary, using novel methodology to isolate components of liability that distinguish schizophrenia, BD, and MDD as well as the pleiotropic component shared between the disorders, we have shown that within BD, clinical heterogeneity was influenced not only by the burden of risk alleles for BD carried by an individual but also by the contribution of alleles that have relatively distinct effects on risk for other traits. It follows that the different phenotypic features within BD may be associated with etiological heterogeneity. Further understanding the basis of this heterogeneity will be critical for obtaining a detailed understanding of the different pathophysiological processes underlying BD, stratifying patients, and developing precision therapeutics.

## Notes

Supplement. **eFigure 1.** Genomic SEM Models Applied to Source GWAS on a SNP-by-SNP Basis to Create Shared and Differentiating gSEM Components **eFigure 2.** Histogram of Continuous BADDS-P Values Across BD and SABP Cases in BDRN Data **eFigure 3.** Histogram of Continuous BADDS-I Values Across BD and SABP Cases in BDRN Data **eFigure 4.** Histogram of Continuous BADDS-M Values Across BD and SABP Cases in BDRN Data **eFigure 5.** Histogram of Continuous BADDS-D Values Across BD and SABP Cases in BDRN Data
eMethods.
**eTable 1.** Sample Size, Year of Publication, Heritability (Observed Scale), and Genetic Correlations of Source GWAS **eTable 2.** Sample Size and Genotyping Platform of BDRN Subsamples **eTable 3.** Samples With Psychosis Data: Sample Size, Genotyping Platform, and Demographic Characteristics of Bipolar Disorder and Schizoaffective Disorder (Bipolar Type) Cases With BADDS-P Data, Divided By Disorder Subtype **eTable 4.** Samples With Psychosis Mood Incongruence Data: Sample Size, Genotyping Platform, and Demographic Characteristics of Bipolar Disorder and Schizoaffective Disorder (Bipolar Type) Cases With BADDS-I Data, Divided By Disorder Subtype **eTable 5.** Pearson’s Correlations Between Source GWAS PRS and gSEM PRS in the BDRN Data **eTable 6.** Polychoric Correlations Between Ordinal Phenotype BADDS Values **eTable 7.** Primary Analysis: Logistic Regression of gSEM PRS on Psychosis and Psychosis Mood Incongruence **eTable 8.** Logistic Regression of gSEM PRS on Psychosis and Psychosis Mood Incongruence **eTable 9.** Primary Analysis of gSEM Component PRS on Mania and Depression Symptom Scores Using Ordinal Logistic Regression **eTable 10.** Ordinal Logistic Regression of gSEM Component PRS on Mania and Depression Symptoms
