# Supplementary material for: Genetic Liabilities Differentiating Bipolar Disorder, Schizophrenia, and Major Depressive Disorder, and Phenotypic Heterogeneity in Bipolar Disorder
Source: JAMA Psychiatry. 2022 Aug 31;79(10):1032–9. doi: 10.1001/jamapsychiatry.2022.2594 (PMC9434480; doi:10.1001/jamapsychiatry.2022.2594)
Supplement: Supplement. — eFigure 1. Genomic SEM Models Applied to Source GWAS on a SNP-by-SNP Basis to Create Shared and Differentiating gSEM Components eFigure 2. Histogram of Continuous BADDS-P Values Across BD and SABP Cases in BDRN Data eFigure 3. Histogram of Continuous BADDS-I Values Across BD and SABP Cases in BDRN Data eFigure 4. Histogram of Continuous BADDS-M Values Across BD and SABP Cases in BDRN Data eFigure 5. Histogram of Continuous BADDS-D Values Across BD and SABP Cases in BDRN Data eMethods. eTable 1. Sample Size, Year of Publication, Heritability (Observed Scale), and Genetic Correlations of Source GWAS eTable 2. Sample Size and Genotyping Platform of BDRN Subsamples eTable 3. Samples With Psychosis Data: Sample Size, Genotyping Platform, and Demographic Characteristics of Bipolar Disorder and Schizoaffective Disorder (Bipolar Type) Cases With BADDS-P Data, Divided By Disorder Subtype eTable 4. Samples With Psychosis Mood Incongruence Data: Sample Size, Genotyping Platform, and Demographic Characteristics of Bipolar Disorder and Schizoaffective Disorder (Bipolar Type) Cases With BADDS-I Data, Divided By Disorder Subtype eTable 5. Pearson’s Correlations Between Source GWAS PRS and gSEM PRS in the BDRN Data eTable 6. Polychoric Correlations Between Ordinal Phenotype BADDS Values eTable 7. Primary Analysis: Logistic Regression of gSEM PRS on Psychosis and Psychosis Mood Incongruence eTable 8. Logistic Regression of gSEM PRS on Psychosis and Psychosis Mood Incongruence eTable 9. Primary Analysis of gSEM Component PRS on Mania and Depression Symptom Scores Using Ordinal Logistic Regression eTable 10. Ordinal Logistic Regression of gSEM Component PRS on Mania and Depression Symptoms [file jamapsychiatry-e222594-s001.pdf]

Supplemental Online Content

Richards AL, Cardno A, Harold G, et al, et al. Genetic liabilities differentiating bipolar disorder, schizophrenia, and major depressive disorder, and phenotypic heterogeneity in bipolar disorder. *JAMA Psychiatry*. Published online August 31, 2022. doi:10.1001/jamapsychiatry.2022.2594

eFigure 1. Genomic SEM Models Applied to Source GWAS on a SNP-by-SNP Basis to Create Shared and Differentiating gSEM Components

eFigure 2. Histogram of Continuous BADDS-P Values Across BD and SABP Cases in BDRN Data

eFigure 3. Histogram of Continuous BADDS-I Values Across BD and SABP Cases in BDRN Data

eFigure 4. Histogram of Continuous BADDS-M Values Across BD and SABP Cases in BDRN Data

eFigure 5. Histogram of Continuous BADDS-D Values Across BD and SABP Cases in BDRN Data

eMethods.

eTable 1. Sample Size, Year of Publication, Heritability (Observed Scale), and Genetic Correlations of Source GWAS

eTable 2. Sample Size and Genotyping Platform of BDRN Subsamples

eTable 3. Samples With Psychosis Data: Sample Size, Genotyping Platform, and Demographic Characteristics of Bipolar Disorder and Schizoaffective Disorder (Bipolar Type) Cases With BADDS-P Data, Divided By Disorder Subtype

eTable 4. Samples With Psychosis Mood Incongruence Data: Sample Size, Genotyping Platform, and Demographic Characteristics of Bipolar Disorder and Schizoaffective Disorder (Bipolar Type) Cases With BADDS-I Data, Divided By Disorder Subtype

eTable 5. Pearson's Correlations Between Source GWAS PRS and gSEM PRS in the BDRN Data

eTable 6. Polychoric Correlations Between Ordinal Phenotype BADDS Values

eTable 7. Primary Analysis: Logistic Regression of gSEM PRS on Psychosis and Psychosis Mood Incongruence

eTable 8. Logistic Regression of gSEM PRS on Psychosis and Psychosis Mood Incongruence

© 2022 Richards AL et al. *JAMA Psychiatry*.

eTable 9. Primary Analysis of gSEM Component PRS on Mania and Depression Symptom Scores Using Ordinal Logistic Regression
eTable 10. Ordinal Logistic Regression of gSEM Component PRS on Mania and Depression Symptoms

This supplementary material has been provided by the authors to give readers additional information about their work.

© 2022 Richards AL et al. *JAMA Psychiatry*.

eFigure 1. Genomic SEM Models Applied to Source GWAS on a SNP-by-SNP Basis to Create Shared and Differentiating gSEM Components

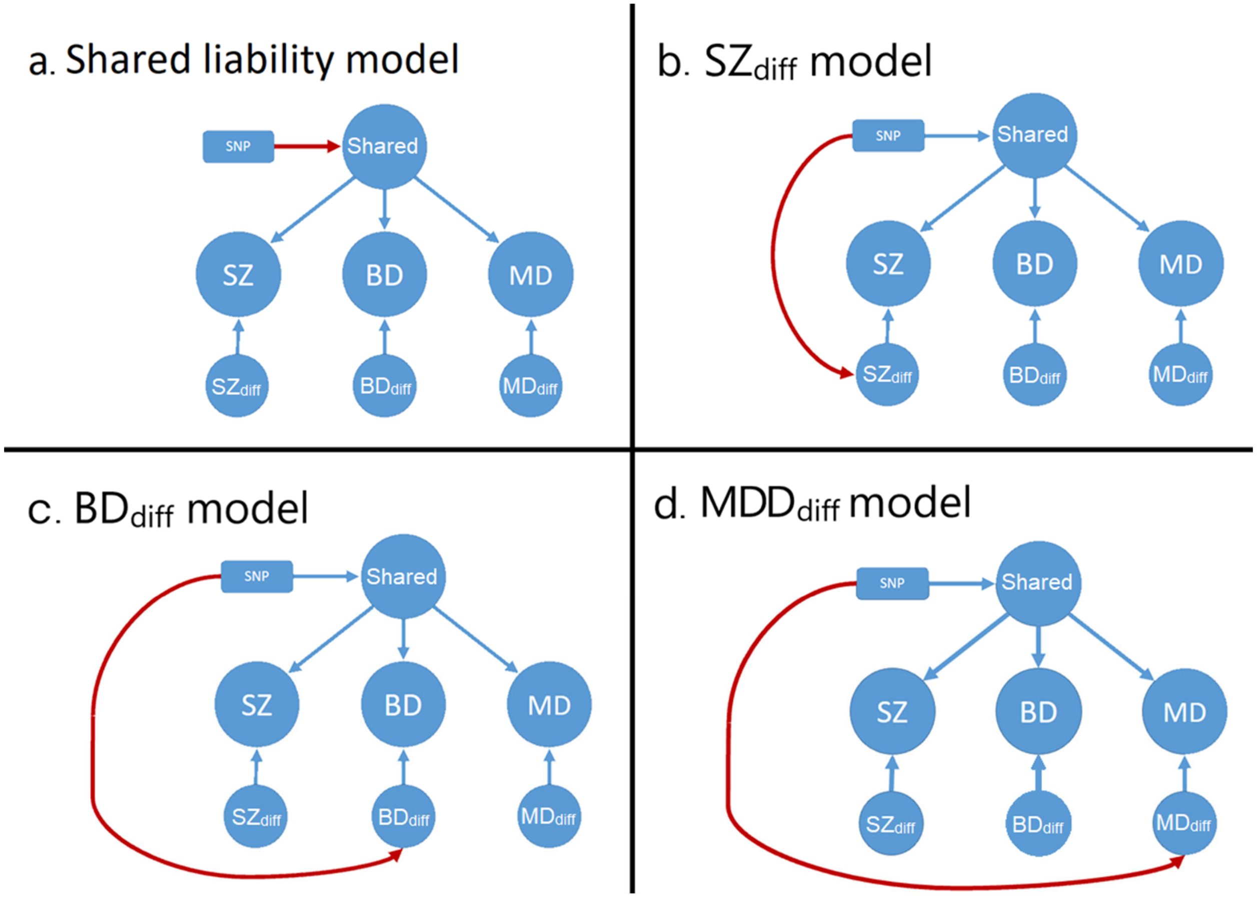

Figure S1a-d. Genomic SEM models applied to source GWAS on a SNP-by-SNP basis to create shared and differentiating gSEM components. SZ, BD and MD indicate the schizophrenia, bipolar disorder and major depressive disorder source GWAS. Shared indicates the liability shared between the three source disorders. SZ_{diff}, BD_{diff} and MD_{diff} indicate the residual genetic liability remaining for each disorder after the shared liability is calculated. Red arrow shows weighting extracted from model.

© 2022 Richards AL et al. *JAMA Psychiatry*.

eFigure 2. Histogram of Continuous BADDS-P Values Across BD and SABP Cases in BDRN Data

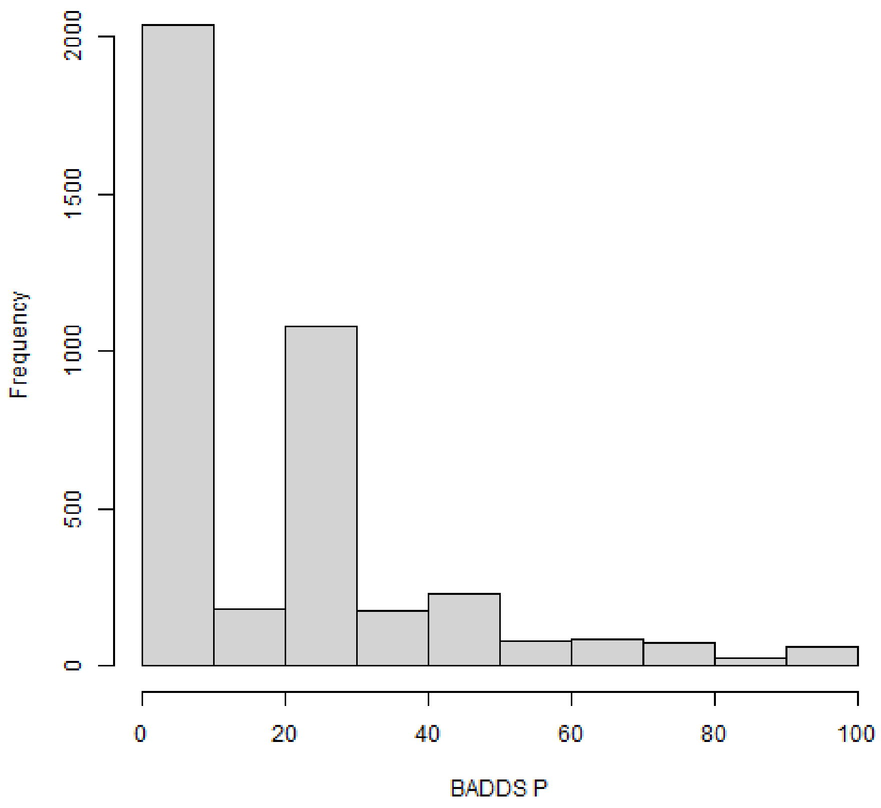

Figure S2. Histogram of continuous BADDS P values across BD and SABP cases in BDRN data.

© 2022 Richards AL et al. *JAMA Psychiatry*.

eFigure 3. Histogram of Continuous BADDS-I Values Across BD and SABP Cases in BDRN Data

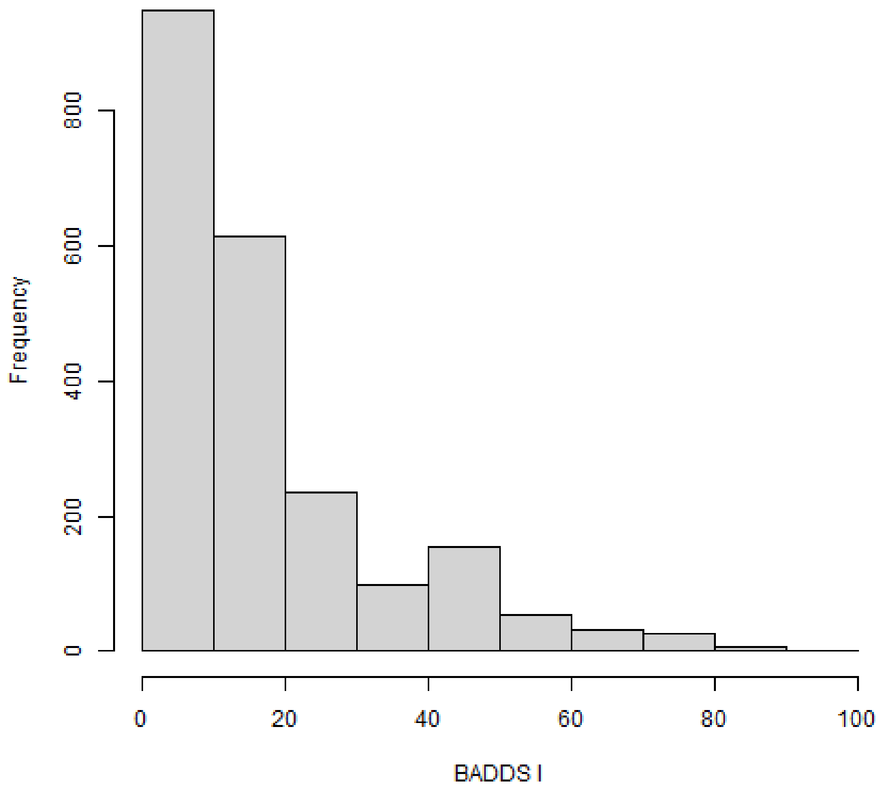

Figure S3. Histogram of continuous BADDS I values across BD and SABP cases in BDRN data.

© 2022 Richards AL et al. *JAMA Psychiatry*.

eFigure 4. Histogram of Continuous BADDS-M Values Across BD and SABP Cases in BDRN Data

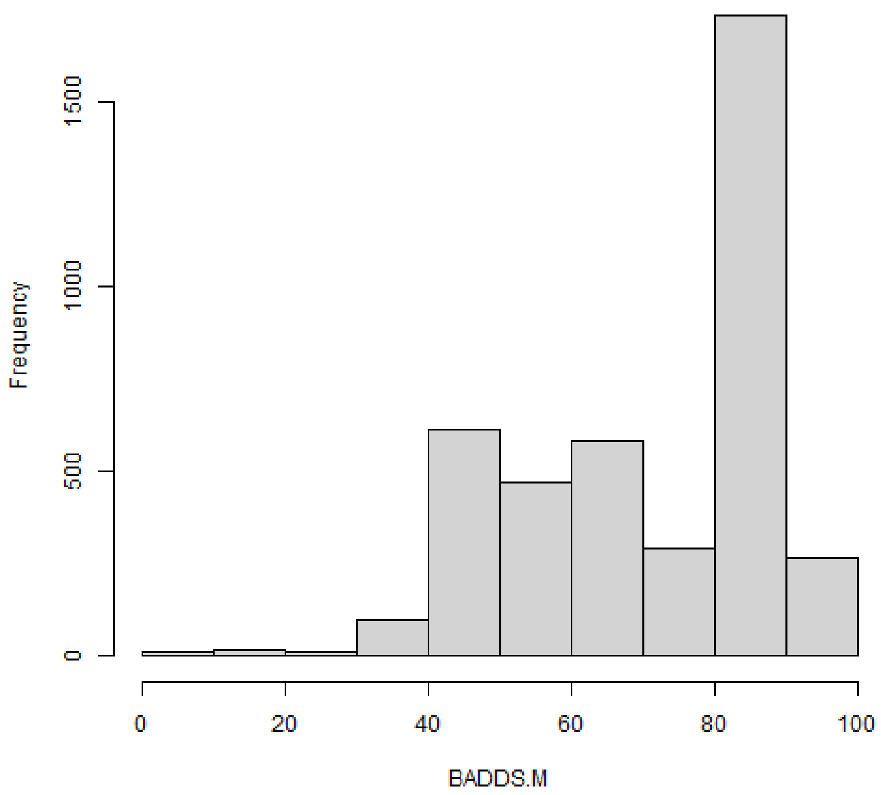

Figure S4. Histogram of continuous BADDS M values across BD and SABP cases in BDRN data.

© 2022 Richards AL et al. *JAMA Psychiatry*.

eFigure 5. Histogram of Continuous BADDS-D Values Across BD and SABP Cases in BDRN Data

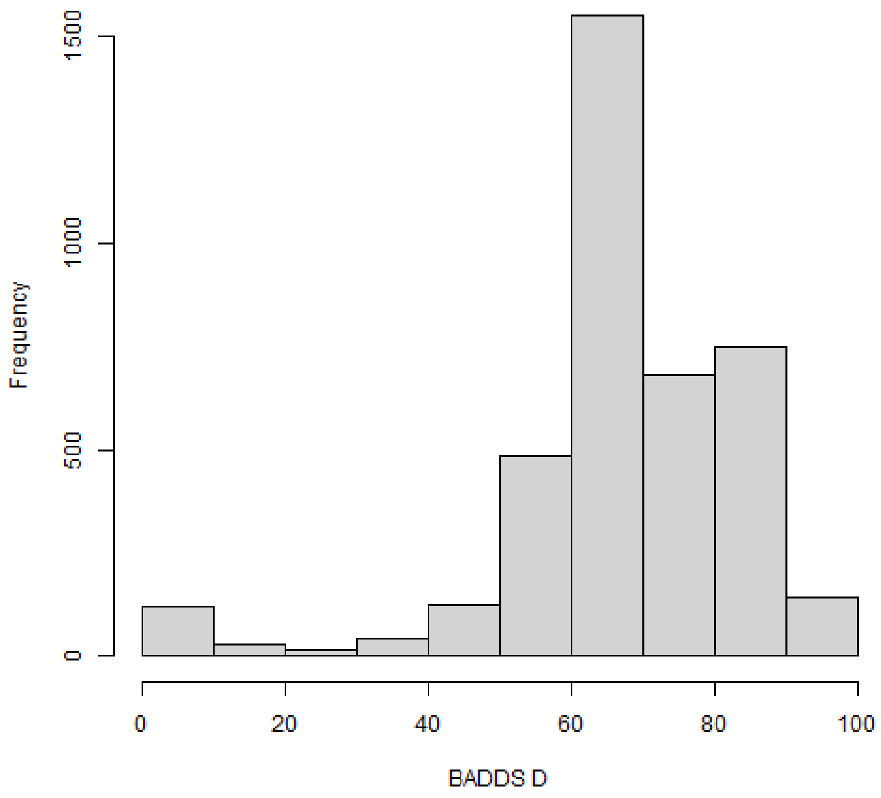

Figure S5. Histogram of continuous BADDS D values across BD and SABP cases in BDRN data.

© 2022 Richards AL et al. *JAMA Psychiatry*.

eMethods.

gSEM method details

gSEM is a two stage process. Initially, the genetic covariance and sampling covariance matrices are estimated from the input GWAS summary statistics files, using a modified version of linkage disequilibrium score regression (LDSC)[1,2]. The sampling covariance matrix includes indices of sample error for each input and also estimates of association between the sample errors of each input. This allows estimation of sample overlap between the input GWAS, permitting gSEM to be used on input GWAS that share samples.

In the second stage, the SEM that the user specifies is applied to each SNP in the dataset in turn. Parameters for this SEM are estimated by minimising the difference between the genetic covariance matrix produced from the model and the genetic covariance matrix estimated in the previous step. For each model, we extracted the loading between the SNP and the term of interest as an estimate of the extent to which that SNP acts through that term (Supplementary Figure S1a-d, weighting extracted shown by red arrow). These loading values were used as weightings for the polygenic risk scores.

The power of gSEM is determined by the ratio between the number of parameters estimated and the sample sizes of the source GWAS, and will often exceed the power of the individual source GWAS. Hence, it is very likely that our power to detect true effects in our phenotypic data exceeds the level of statistical adequacy (B=0.8) and is likely to be at least comparable to the source GWAS.

SNP-based heritabilities (SNP_{h2}) were calculated for gSEM components using the base model of linkage disequilibrium score regression (LDSC)[1,3]. We report heritability on the observed scale because the population prevalences of the constructs underlying gSEM components are unknown, precluding conversion to liability scale.

lavaan code for four models

Shared model

F1 =~ SZ + BD + MDD

F1 ~ SNP

SZ_{diff} model

F1 =~ SZ + BD + MDD

F1 + SZ ~ SNP

© 2022 Richards AL et al. *JAMA Psychiatry*.

BD_{diff} model

F1 =~ SZ + BD + MDD

F1 + BD ~ SNP

MDD_{diff} model

F1 =~ SZ + BD + MDD

F1 + MDD ~ SNP

References

1. Bulik-Sullivan BK, Loh PR, Finucane HK, et al. LD Score regression distinguishes confounding from polygenicity in genome-wide association studies. *Nat Genet*. Mar 2015;47(3):291-5. doi:10.1038/ng.3211
2. Grotzinger AD, Rhemtulla M, de Vlaming R, et al. Genomic structural equation modelling provides insights into the multivariate genetic architecture of complex traits. *Nat Hum Behav*. May 2019;3(5):513-525. doi:10.1038/s41562-019-0566-x
3. Bulik-Sullivan B, Finucane HK, Anttila V, et al. An atlas of genetic correlations across human diseases and traits. *Nat Genet*. Nov 2015;47(11):1236-41. doi:10.1038/ng.3406

© 2022 Richards AL et al. *JAMA Psychiatry*.

eTable 1. Sample Size, Year of Publication, Heritability (Observed Scale), and Genetic Correlations of Source GWAS

Source GWAS	Sample size	Heritability (SNP_{h2})	Standard error of heritability	Year of publication	Reference	Genetic correlations (rg)	
						SZ	BD
**SZ (European samples)**	130644	0.3499	0.0122	2022	doi: 10.1038/s41586-022-04434-5		
**BD (European samples)**	398495	0.259	0.0116	2021	doi: 10.1101/2020.09.17.20187054	0.654	
**MDD (excluding 23andMe)**	173005	0.0704	0.0044	2018	doi: 10.1038/s41588-018-0090-3	0.382	0.487

© 2022 Richards AL et al. *JAMA Psychiatry*.

eTable 2. Sample Size and Genotyping Platform of BDRN Subsamples

	N cases	N controls	Chip
**Wellcome Trust Case-Control Consortium (WTCCC)**	1278	2934	GeneChip 500K Mapping Array Set (Affymetrix)
**Bipolar Disorder Research Network Wave 1 (ICCBD)**	2295	2784	Omni Express (Illumina)
**Bipolar Disorder Research Network Wave 2**	887	0	PsychChip (Illumina)
**Total**	4460	5718	

© 2022 Richards AL et al. *JAMA Psychiatry*.

eTable 3. Samples With Psychosis Data: Sample Size, Genotyping Platform, and Demographic Characteristics of Bipolar Disorder and Schizoaffective Disorder (Bipolar Type) Cases With BADDS-P Data, Divided By Disorder Subtype

Subtype	N with non-missing BADDS P	Sex (% female)	N on Affymetrix 500K GeneChip	N on Omni Express	N on PsychChip	Mean age onset / impairment	Mean age at interview
BP1	2688	67.9	906	1349	433	22.62	45.80
BP2	1129	68.9	94	711	324	20.08	44.06
BP NOS	71	70.4	39	0	32	22.96	43.55
SABP	136	58.1	40	76	20	20.20	43.89
All	4024	67.9	1079	2136	809	21.83	45.21

© 2022 Richards AL et al. *JAMA Psychiatry*.

eTable 4. Samples With Psychosis Mood Incongruence Data: Sample Size, Genotyping Platform, and Demographic Characteristics of Bipolar Disorder and Schizoaffective Disorder (Bipolar Type) Cases With BADDS-I Data, Divided By Disorder Subtype

Subtype	N with non-missing BADDS I	Sex (% female)	N on Affymetrix 500K GeneChip	N on Omni Express	N on PsychChip	Mean age onset / impairment	Mean age at interview
BP1	1871	69.7	671	898	302	22.56	45.02
BP2	121	84.3	28	71	22	19.73	45.50
BP NOS	15	86.7	12	0	3	25.07	44.93
SABP	151	59.6	40	84	27	20.21	44.26
All	2158	69.9	751	1053	354	22.25	44.99

Supplementary Table S4. Samples with psychosis mood incongruence data: sample size, genotyping platform and demographic characteristics of bipolar disorder and schizoaffective disorder (bipolar type) cases with BADDS I data, divided by disorder subtype. N=number.

© 2022 Richards AL et al. *JAMA Psychiatry*.

eTable 5. Pearson's Correlations Between Source GWAS PRS and gSEM PRS in the BDRN Data

	Source SZ GWAS	Source BD GWAS	Source MDD GWAS	Shared	SZ_{diff}	BD_{diff}	MDD_{diff}
**Source SZ GWAS**		0.31	0.13	0.79	0.46	-0.18	-0.30
**Source BD GWAS**	3.88E-122		0.15	0.66	-0.37	0.67	-0.23
**Source MD GWAS**	1.10E-22	1.84E-28		0.40	-0.43	-0.23	0.71
**Shared**	0	0	5.46E-206		-0.01	0.09	-0.07
**SZ_{diff}**	1.02E-288	5.50E-175	7.23E-242	0.54		-0.39	-0.52
**BD_{diff}**	1.01E-38	0	4.82E-67	2.60E-12	1.95E-195		-0.37
**MDD_{diff}**	2.80E-116	5.37E-67	0	8.28E-08	0	4.85E-172	

Supplementary Table S5. Pearson's correlations between source GWAS PRS and gSEM PRS in the BDRN data. r values are above diagonal, correlation p-values are below diagonal. Red cells indicate positive correlations, blue cells indicate negative correlations.

© 2022 Richards AL et al. *JAMA Psychiatry*.

eTable 6. Polychoric Correlations Between Ordinal Phenotype BADDS Values

	Psychosis	Incongruence	Mania	Depression
**Psychosis**		0.38	0.61	0.07
**Incongruence**	<1e-5		0.14	0.04
**Mania**	<1e-5	<1e-5		-0.06
**Depression**	<1e-5	0.08	<1e-5	

Supplementary Table S6. Polychoric correlations between ordinal phenotype BADDS values. r values are above diagonal, bootstrap correlation p-values are below diagonal (number of iterations = 100000). Red cells indicate positive correlations, blue cells indicate negative correlations. Note that the correlation between psychosis and incongruence may be due to structural factors (incongruence is only rated in samples with BADDS P ≥ 10).

© 2022 Richards AL et al. *JAMA Psychiatry*.

eTable 7. Primary Analysis: Logistic Regression of gSEM PRS on Psychosis and Psychosis Mood Incongruence

		Logistic (primary analysis)			Ordinal logistic (secondary analysis)		
**Score**	**Phenotype**	**Beta**	**SE**	**P**	**Beta**	**SE**	**P**
Shared	Psychosis	0.054	0.007	2.33E-13	0.221	0.029	1.45E-14
SZ_{diff}	Psychosis	0.028	0.007	0.0001	0.107	0.028	0.0001
BD_{diff}	Psychosis	0.020	0.007	0.006	0.091	0.028	0.001
MDD_{diff}	Psychosis	-0.052	0.007	1.26E-12	-0.217	0.028	1.74E-14
Shared	Incongruence	0.009	0.010	0.348	0.069	0.038	0.072
SZ_{diff}	Incongruence	0.029	0.010	0.003	0.105	0.037	0.005
BD_{diff}	Incongruence	-0.014	0.010	0.140	-0.070	0.038	0.065
MDD_{diff}	Incongruence	-0.016	0.010	0.112	-0.041	0.039	0.284

Supplementary Table S7. Primary analysis: logistic regression of gSEM PRS on psychosis and psychosis mood incongruence, thresholded (BADDS P ≥ 10, BADDS I ≥ 20) to make them dichotomous. Secondary analyses use ordinal logistic regression. Both analyses covary for age at interview, genotyping platform and 10 PCs. The Bonferroni correction threshold for 16 independent primary tests (4 gSEM component PRS, 4 phenotypes) is 0.0031 but we note that this is excessively conservative given the correlations between gSEM components (Table S5) and also those between the phenotypes (Table S6). SZ_{diff}, BD_{diff} and MDD_{diff} indicate the differentiating PRS for schizophrenia, bipolar disorder and major depressive disorder respectively.

© 2022 Richards AL et al. *JAMA Psychiatry*.

eTable 8. Logistic Regression of gSEM PRS on Psychosis and Psychosis Mood Incongruence

		Logistic			Ordinal logistic		
**Score**	**Phenotype**	**Beta**	**SE**	**P**	**Beta**	**SE**	**P**
Shared	Psychosis	0.054	0.008	5.42E-13	0.222	0.029	4.47E-14
SZ_{diff}	Psychosis	0.027	0.007	0.0002	0.096	0.028	0.0007
BD_{diff}	Psychosis	0.021	0.007	0.004	0.103	0.029	0.0003
MDD_{diff}	Psychosis	-0.053	0.007	1.58E-12	-0.215	0.029	9.90E-14
Shared	Incongruence	0.010	0.010	0.325	0.071	0.040	0.077
SZ_{diff}	Incongruence	0.028	0.010	0.005	0.091	0.039	0.020
BD_{diff}	Incongruence	-0.012	0.010	0.230	-0.051	0.039	0.195
MDD_{diff}	Incongruence	-0.017	0.010	0.105	-0.044	0.040	0.277

Supplementary Table S8. Logistic regression of gSEM PRS on psychosis and psychosis mood incongruence, thresholded (BADDS P ≥ 10, BADDS I ≥ 20) to make them dichotomous, excluding samples with schizoaffective disorder (SZA). Secondary analyses use ordinal logistic regression. Both analyses covary for age at interview, genotyping platform and 10 PCs. SZ_{diff}, BD_{diff} and MDD_{diff} indicate the differentiating PRS for schizophrenia, bipolar disorder and major depressive disorder respectively.

© 2022 Richards AL et al. *JAMA Psychiatry*.

eTable 9. Primary Analysis of gSEM Component PRS on Mania and Depression Symptom Scores Using Ordinal Logistic Regression

Score	Phenotype	Beta	SE	P
Shared	Mania	0.288	0.028	3.04E-25
SZ_{diff}	Mania	0.084	0.027	0.002
BD_{diff}	Mania	0.142	0.027	1.99E-07
MDD_{diff}	Mania	-0.217	0.028	2.84E-15
Shared	Depression	-0.003	0.028	0.910
SZ_{diff}	Depression	0.024	0.028	0.385
BD_{diff}	Depression	-0.111	0.028	7.06E-05
MDD_{diff}	Depression	0.069	0.028	0.013

Supplementary Table S9. Primary analysis of gSEM component PRS on mania and depression symptom scores using ordinal logistic regression. Covaried for age at interview, genotyping platform and 10 PCs. The Bonferroni correction threshold for 16 independent primary tests (4 gSEM component PRS, 4 phenotypes) is 0.0031 but we note that this is excessively conservative given the correlations between gSEM components (Table S5) and also those between the phenotypes (Table S6). SZ_{diff}, BD_{diff} and MDD_{diff} indicate the differentiating PRS for schizophrenia, bipolar disorder and major depressive disorder respectively.

© 2022 Richards AL et al. *JAMA Psychiatry*.

eTable 10. Ordinal Logistic Regression of gSEM Component PRS on Mania and Depression Symptoms

Score	Phenotype	Beta	SE	P
Shared	Mania	0.291	0.028	1.09E-24
SZ_{diff}	Mania	0.088	0.028	0.002
BD_{diff}	Mania	0.149	0.028	9.16E-08
MDD_{diff}	Mania	-0.223	0.028	2.31E-15
Shared	Depression	-0.006	0.029	0.834
SZ_{diff}	Depression	0.023	0.028	0.426
BD_{diff}	Depression	-0.121	0.029	2.37E-05
MDD_{diff}	Depression	0.077	0.029	0.007

Supplementary Table S10. Ordinal logistic regression of gSEM component PRS on mania and depression symptoms, using raw BADDS M and BADDS D scores, excluding samples with schizoaffective disorder. Covaried for age at interview, genotyping platform and 10 PCs. SZ_{diff}, BD_{diff} and MDD_{diff} indicate the differentiating PRS for schizophrenia, bipolar disorder and major depressive disorder respectively.

© 2022 Richards AL et al. *JAMA Psychiatry*.
